# Functional connectivity of the amygdala and the antidepressant and antisuicidal effects of repeated ketamine infusions in major depressive disorder

**DOI:** 10.3389/fnins.2023.1123797

**Published:** 2023-02-02

**Authors:** Haiyan Liu, Chengyu Wang, Xiaofeng Lan, Weicheng Li, Fan Zhang, Ling Fu, Yanxiang Ye, Yuping Ning, Yanling Zhou

**Affiliations:** ^1^Department of Child and Adolescent Psychiatry, Affiliated Brain Hospital of Guangzhou Medical University, Guangzhou, China; ^2^Key Laboratory of Neurogenetics and Channelopathies of Guangdong Province and the Ministry of Education of China, The Second Affiliated Hospital of Guangzhou Medical University, Guangzhou, China; ^3^Guangdong Engineering Technology Research Center for Translational Medicine of Mental Disorders, Guangzhou, China; ^4^Department of Psychology, The First School of Clinical Medicine, Southern Medical University, Guangzhou, China

**Keywords:** major depressive disorder, ketamine, amygdala, functional connectivity, antidepressant

## Abstract

**Background:**

Dysfunction of the amygdala is the core pathogenesis of major depressive disorder (MDD). However, it remains unclear whether ketamine treatment could modulate characteristics of amygdala-related networks. We aimed to explore the relationship between changes in the resting-state functional connectivity (RSFC) of the amygdala and the treatment of ketamine in MDD patients and to identify important neuroimaging predictors of treatment outcome.

**Methods:**

Thirty-nine MDD patients received six subanesthetic dose infusions of ketamine. Depressive and suicidal symptoms were assessed and magnetic resonance imaging (MRI) scans were performed before and after six ketamine infusions. Forty-five healthy controls also underwent once MRI scans. Seed-based RSFC analyses were performed, focusing on the bilateral amygdala.

**Results:**

After ketamine treatment, the RSFC between the left amygdala (LA) and the left medial superior frontal gyrus (mSFG) of MDD patients enhanced significantly, and this change was positively correlated with the reduction in depressive symptoms (*r* = 0.40, *p* = 0.012). The combination baseline RSFC of LA – right putamen and right amygdala (RA) – right putamen was related to the antidepressant and antisuicidal effects of ketamine. The combination baseline RSFC of LA – right putamen and RA – right putamen could predict the ineffective antidepressant (AUC = 0.739, *p* = 0.011) and antisuicidal effects of ketamine (AUC = 0.827, *p* = 0.001).

**Conclusion:**

Ketamine can regulate the relevant circuits of amygdala and mSFG, and the baseline RSFC between bilateral amygdala and right putamen may be a predictor of the response of ketamine’s antidepressant and antisuicidal treatment.

**Clinical trial registration:**

https://www.chictr.org.cn/showproj.aspx?proj=20875, identifier ChiCTR-OOC-17012239.

## 1. Introduction

Major depressive disorder (MDD) is a major contributor to the global burden of disease, due to its high incidence of disability and suicide (Ferrari et al., 2013). However, a high proportion of patients have poor outcomes after receiving the current evidence-based treatments: less than half recover within the first 3 months of treatment (Trivedi et al., 2006), and nearly one-third do not respond to two or more traditional antidepressant medications (McGrath et al., 2006; Mrazek et al., 2014).

Ketamine, a novel antidepressant, is an N-methyl-D-aspartate (NMDA) receptor antagonist that has attracted widespread attention for its rapid antidepressant effects. Evidence has demonstrated that subanesthetic doses of ketamine could rapidly and effectively improve depressive symptoms and suicidal ideation in MDD patients (Wilkinson et al., 2018; Zheng et al., 2018; Marcantoni et al., 2020). Repeated use of ketamine over 2–4 weeks may be more effective and longer lasting than a single dose (Aan et al., 2010; Murrough et al., 2013; Zheng et al., 2018, 2019). However, some patients still failed to respond to ketamine treatment. For example, our previous study reported nearly 40% did not respond to six infusions of ketamine (Zheng et al., 2019). Therefore, using neuroimaging to determine predictor of response to ketamine treatment and the neural mechanism of its antidepressant and antisuicidal effects is crucially important.

Our previous study found that the volume of the left amygdala (LA) increased after six ketamine treatments, corresponding to an improvement in depressive symptoms (Zhou et al., 2020). As the amygdala is the main area responsible for emotion perception and generation (Davis and Whalen, 2001; Phelps and LeDoux, 2005), further analysis of the amygdala using functional imaging, is needed to determine whether its resting-state functional connectivity (RSFC) would be involved in ketamine’s antidepressant action.

The amygdala is involved in key symptoms of MDD, not only related to the regulation of emotions and sensory processing but also to the processing of visceral information related to emotional stimuli (Price, 2003). A large sample study found that amygdala voxels had decreased RSFC with the orbitofrontal cortex, temporal lobe areas, including the temporal pole, inferior temporal gyrus and the parahippocampal gyrus in MDD patients compared to healthy controls (Cheng et al., 2018). Another meta-analysis showed that in MDD patients, abnormal RSFC of the amygdala occurred mainly in the affective network, including strengthened RSFC with the right hippocampus/parahippocampus and bilateral ventromedial orbitofrontal cortex and weakened RSFC with the bilateral insula and left caudate (Tang et al., 2018). The alteration in the RSFC of the amygdala is also associated with the risk of suicide. Emotional disorder patients with suicidal ideation or those who have attempted suicide exhibited abnormal RSFC between the amygdala and regions such as the parahippocampal area, paracentral lobule/precuneus, insula, middle temporal gyrus, and superior orbitofrontal area compared to those without suicidal ideation/suicide attempts (Kang et al., 2017; Zhang et al., 2020).

In addition, antidepressant treatment was found to associate with changes in the RSFC of the amygdala. Fluoxetine treatment was associated with significantly strengthened RSFC between the amygdala and right frontal and cingulate cortex, striatum, and thalamus in MDD patients (Chen et al., 2008). Transcutaneous vagus nerve stimulation could reduce depressive symptoms, accompanied by strengthened RSFC in the right amygdala (RA) and left dorsolateral prefrontal cortex (PFC) (Liu et al., 2016). However, up to now it is not clear whether the amygdala RSFC play a role in modulating ketamine’s antidepressant and antisuicide effects.

Herein, we set the amygdala as a region of interest (ROI) and applied a voxel-based RSFC analysis to investigate alterations in RSFC between the mentioned above seed point and the whole brain after six infusions of ketamine treatment in MDD patients. Then we investigated the relationship between these changes and reductions in depressive symptoms and suicidal ideation after ketamine treatment. Additionally, the receiver operating characteristic (ROC) curve analysis was used to examine the baseline amygdala RSFC could predict the antidepressant and antisuicidal effects of ketamine.

## 2. Methods

### 2.1. Participants

The data came from a clinical trial that explored the antidepressant effect of repeated ketamine infusions on MDD patients (clinical trial No. ChiCTR-OOC-17012239). Patients who met the following criteria (regardless of sex) were included in the study: (1) 18–65 years old; (2) diagnosed with MDD without psychotic features according to the structured clinical interview for Diagnostic and Statistical Manual of Mental Diseases-5 (DSM-5); (3) failed to respond to at least two suitable antidepressant agents with adequate dosage or exhibited suicidal ideation [confirmed by the Beck Scale for Suicidal Ideation (SSI) – Part I, with a score ≥2 at screening]; (4) total score of 17 items of Hamilton Depression Rating Scale (HAMD-17) ≥17; (5) no current pregnancy or lactation; (6) without serious medical or nervous system diseases and substance dependence; (7) no metal implants or other magnetic resonance imaging (MRI) contraindications; and (8) underwent resting-state functional MRI (rs-fMRI) scans at baseline and follow-up.

Forty-five healthy controls (HCs) were recruited from the community. All HCs were in a healthy state without any previous or current mental illness or drug abuse or dependence. At the same time, there were no contraindications to MRI.

All research procedures were conducted in accordance with the Declaration of Helsinki ethical principles and approved by the Review Committee of the Brain Hospital Affiliated to Guangzhou Medical University. All subjects had provided written informed consent before participation.

### 2.2. Procedures

All MDD patients received a 40-min open-label infusion of ketamine (0.5 mg/kg) three times a week for 2 weeks. The details of our methods have been described in our previous studies (Zheng et al., 2018; Zhou et al., 2018a,b). Hemodynamics and clinical status were monitored during this period. The MDD patients were not restricted from using psychiatric drugs throughout the study, but were only eligible to use if they took a stable dosage of antidepressants for at least 4 weeks before entering the study and continuing to receive the same regimen and dose throughout the study.

MDD patients were assessed for clinical symptoms and underwent rs-fMRI scans 24 h prior to ketamine infusion (T0) and 24 h after the sixth ketamine infusion (T1). HCs underwent only one rs-fMRI scan.

### 2.3. Rating scales

The severity of depressive symptoms was assessed with the HAMD-17; the higher their score on this scale, the more severe the depressive symptoms were. The SSI scale was used to assess suicidal ideation and which was consisted of 19 items administered by a clinician. We utilized the first five items of the SSI (SSI-5), which included hope to live, hope to die, reasons for living or dying, desire to actively attempt suicide, and passive suicidal thoughts. The higher score of SSI-5 indicated a higher risk of suicide. The interrater reliability of the clinicians who administered the HAMD-17 was assessed; the intraclass correlation coefficients were >0.9.

The reduction rate (ΔHAMD-17% and ΔSSI-5%) was used to index the antidepressant and antisuicide effect of ketamine. The reduction rate was calculated with the following equation: pre-treatment score minus post-treatment score, then divided by the pre-treatment score, and finally multiplied by 100%. Responders were defined as having a reduction rate in HAMD-17 score or SSI-5 score ≥50%.

### 2.4. MRI acquisition

All imaging data were acquired by the Philips Achieva X-series 3T scanner with an eight-channel phased-array head coils. The gradient-echo echo-planar imaging (GRE-EPI) sequence was used to acquire blood oxygen level dependent (BOLD) images with an echo time of 30 ms, repetition time of 2,000 ms, flip angle of 90°, filed of view of 220 mm × 220 mm^2^, matrix of 64 × 64, slice thickness of 4 mm, slice gap of 0.6 mm. The functional run lasted 8 min and 240 volumes acquired. Total of 33 transverse interleaved slices covered the whole brain. During the scan, the subjects were instructed to remain still with their eyes closed, avoid systematic thinking and remain awake.

### 2.5. Image preprocessing

All rsfMRI data was preprocessed using the Data Processing and Analysis of Brain Imaging (DPABI, version 5.0)^1^. The main steps were as follows: (1) removing the first 10 volumes; (2) slice timing; (3)realigning (subjects who had excessive head motion >2.0 mm translation and/or >2.0° rotation during the scan were excluded); (3) spatially normalizing by using EPI templates; (4) smoothing using a 4-mm full width at half maxima (FWHM) isotropic Gaussian kernel; (5) removing linear and quadratic trends; (6) regressing out head motion effects using the Friston 24-parameter model, the white matter and cerebrospinal fluid. Moreover, the global signal was regressed in the whole brain analysis at the same time (Fox et al., 2009; Engman et al., 2016); (7) temporal band-pass filtering (0.01–0.1 Hz); and (8) “scrubbed” one time points before and one time points after bad images, whose frame displacement (FD) >0.5.

### 2.6. Definition of ROI and RSFC analyses

The bilateral amygdala was defined as seed-based two ROI according to the automated anatomical labels (Tzourio-Mazoyer et al., 2002). Then, whole-brain voxel-level RSFC of each amygdala was mapped for all subjects. The main steps were as follows: (1) calculating the mean time series of each amygdala; (2) calculating the Pearson correlation coefficients between the mean time series of each amygdala and that of each voxel in the rest of the brain; and (3) converting each correlation coefficient *z* values using Fisher’s *z* transformation to improve normality.

## 3. Statistical analysis

### 3.1. Baseline demographic characteristics

Independent-sample *t*-tests and χ^2^ tests were conducted to determine whether the MDD and HC groups differed in age and sex with the Statistical Package for Social Sciences (SPSS 25.0). All significance levels were set at *p* < 0.05.

### 3.2. Imaging analysis

The DPABI (version 5.0) toolbox was used to obtain the RSFC from the bilateral amygdala to the whole-brain of HCs, pre-treatment and post-treatment MDD patients. The difference in the mean time course of the rs-fMRI scans before and after ketamine treatment was defined as the change in the RSFC of the amygdala.

First, the statistical parametric mapping version 12 (SPM12),^2^ was used to apply two sample *t*-test comparing the RSFC images between the HCs and pre-treatment MDD groups, controlling for age, sex, and head motion. If aberrant RSFC was found in MDD patients, the RSFC of the amygdala in this region was extracted for correlation analysis with the HAMD-17 score, SSI-5 score.

Second, to investigate the relationship between the changes in abnormal RSFC of amygdala and the improvements in clinical symptoms after ketamine treatment, paired *t*-test was applied with SPM12 to compare the RSFC of pre-treatment and pro-treatment MDD patients, with head motion as a covariate. If the change in the RSFC of the amygdala was found in post-treatment patients, we would extract the above RSFC for correlation analysis with the reduction rate of HAMD-17 and SSI-5 scores.

Third, the correlation analysis between abnormal amygdala RSFC at baseline and the reduction rate of depressive symptoms and suicidal symptoms after ketamine treatment were conducted, and ROC curves were performed to determine whether the abnormal RSFC of the amygdala at baseline could predict the treatment effect of ketamine.

All resulting group-level analyses had a threshold of *p* < 0.05 [cluster-corrected using the familywise error rate (FWE) with a height threshold of *p* < 0.001]. All Images were displayed through the DPABI Viewer.

## 4. Results

### 4.1. Baseline characteristics

A total of 45 MDD patients were enrolled in the study. Of these, one exhibited serious artifacts in the rs-fMRI scan, and another five were excluded due to head movement during the scans. Therefore, 39 MDD patients were included in the final analysis.

The two groups (HCs and MDD) exhibited a significant difference in age; specifically, the mean age of the MDD group was higher than that of the HC group (36.5 ± 12.1 vs. 31.4 ± 8.0, *p* = 0.031). The baseline characteristics were shown in Table 1.

**TABLE 1:** Baseline characteristics of participants.

Characteristic	MDD	HCs	*t*/χ^2^	*P*-value
	(n = 39)	(n = 45)		
Age (years)	36.5 ± 12.1	31.4 ± 8.0	–2.206	0.031
Gender (% female)^a^	24 (61.5%)	27 (60.0%)		0.886
Education (years)	11.8 ± 3.3			
BMI (kg/m^2^)	23.0 ± 3.2			
Duration of illness (months)	84.1 ± 80.0			
Age of onset (years)	29.0 ± 11.4			
First episode (yes)	15 (38.5%)			
Psychiatric comorbidity	6 (15.4%)			
Current smoking (yes)	5 (12.8%)			
Current drinking (yes)^b^	1 (2.6%)			
Baseline HAMD-17 score	23.2 ± 4.6			
Baseline SSI-5 score	9.0 ± 3.5			
Use of drugs				
Antidepressant dose (mg/day)	44.4 ± 24.1			
Benzodiazepines	21 (53.8%)			
Antipsychotics	25 (64.1%)			

MDD, major depressive disorder; HCs, healthy controls; BMI, body mass index; HAMD-17, 17-item Hamilton Depression Rating Scale; SSI-5, the first five items of Baker Suicide Scale.

^a^χ^2^ test of continuity correction.

^b^Drinking behavior without alcohol abuse or alcohol dependence.

### 4.2. Abnormal amygdala RSFCs in MDD patients

**FIGURE 1:**
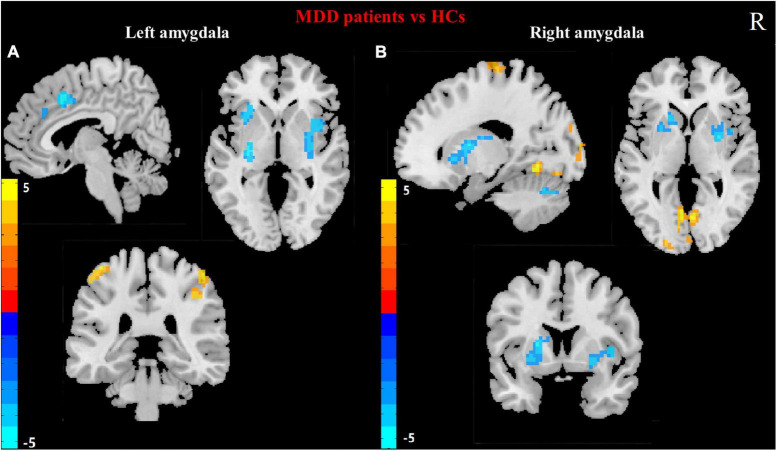
Differences in left **(A)** and right **(B)** amygdala RSFC between MDD patients and HCs. (Two sample *t*-test, voxel- level *p* < 0.001, corrected by FWE). Color bar represents *t*-values. R: right.

For each of the two *a priori*-defined amygdala seeds, we assessed and compared its whole-brain RSFCs in MDD patients and HCs. Compared to HCs, MDD patients displayed hypoconnectivity between LA and bilateral putamen, right MCC and left insula. In addition, MDD patients showed hyperconnectivity between the LA and the bilateral posterior central gyrus (PCG) (Table 2 and Figure 1A).

**TABLE 2:** Abnormal amygdala RSFCs in pre-treatment MDD patients compared to HCs.

ROI	Brain regions (AAL)	Side	Cluster size	MNI coordinates (peak)^a^			*t*-Value	pFWE-corr
				*x*	*y*	*z*		
**LA**	MDD_T0_ > HCs							
	PCG	L	95	−42	−36	63	4.84	0.001
	PCG	R	62	48	−30	57	5.18	0.006
	MDD_T0_ < HCs							
	MCC	R	162	6	15	42	5.13	<0.001
	Putamen	R	162	30	−9	9	4.69	<0.001
	Putamen	L	65	−30	−15	0	5.31	0.005
	Insula	L	83	−33	−15	3	4.82	0.001
**RA**	MDD_T0_ > HCs							
	Calcarine gyrus	L	48	0	−90	−6	4.15	0.023
	Lingual gyrus	L	241	−6	−66	−3	5.37	<0.001
	MOG	L	58	−21	−93	0	3.88	0.009
	Paracentral lobule	L	44	−15	−24	75	3.92	0.034
	MDD_T0_ < HCs							
	Cerebellum_6	L	179	−12	−66	−24	5.43	<0.001
	Putamen	R	88	30	0	0	4.81	0.001
	Undefined*	L	132	−18	3	12	4.66	<0.001

RSFC, resting-state functional connectivity; MDD, major depressive disorder; HCs, healthy controls; ROI, region of interest; AAL, anatomical automatic labeling; FWE, family-wise error rate; LA, left amygdala; RA, right amygdala; PCG, posterior central gyrus; MCC, mid-cingulate cortex; MOG, middle occipital gyrus; L, left; R, right.

^a^*x, y, z* = MNI (Montreal Neurological Institute) coordinates of significant effects.

*The peak point of the cluster did not fall in the AAL division area, which contains part of the putamen.

Compared to HCs, the hypoconnectivity was showed between the RA and left cerebellum_6 as well as the RA and the right putamen in MDD patients. Moreover, MDD patients displayed hyperconnectivity between RA and left calcarine gyrus, left lingual, the left middle occipital gyrus (MOG) and left paracentral lobule (Table 2 and Figure 1B).

### 4.3. Correlation of abnormal amygdala RSFC with depressive symptoms and suicidal ideation in MDD patients at baseline

**FIGURE 2:**
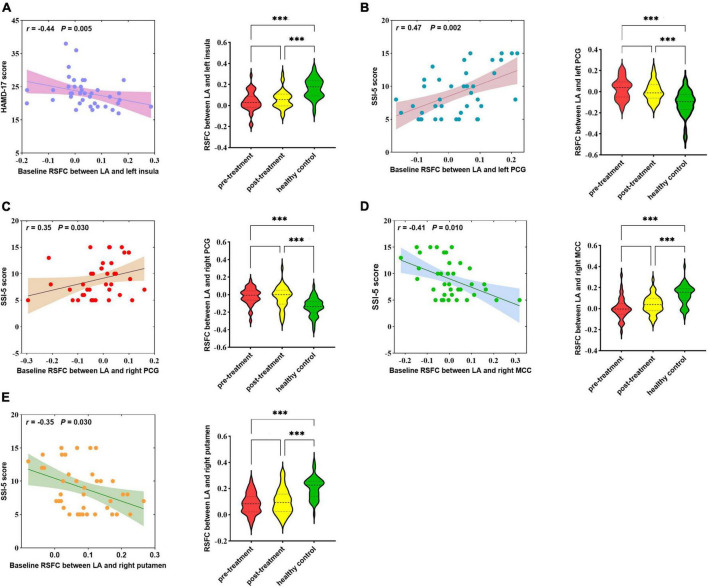
Correlation between abnormal RSFCs of bilateral amygdala and HAMD-17 score **(A)** or SSI-5 score **(B–E)** in MDD patients at baseline. RSFC, resting-state functional connectivity; MDD, Major depressive disorder; HCs, healthy controls; LA, left amygdala; PCG, posterior central gyrus; MCC, mid-cingulate cortex; MOG, middle occipital gyrus. ^***^P < 0.001.

At baseline, showed as Figure 2, HAMD-17 score was negatively correlated with RSFC in the LA and left insula (*r* = −0.44, *p* = 0.005). SSI-5 score was positively correlated with the abnormal RSFCs of LA and left PCG (*r* = 0.47, *p* = 0.002) as well as LA and right PCG (*r* = 0.35, *p* = 0.030). SSI-5 score was also negatively correlated with abnormal RSFC in the LA and right MCC (*r* = −0.41, *p* = 0.010), the LA and the right putamen (*r* = −0.35, *p* = 0.030).

No abnormal RSFC was found in the RA associated with HAMD-17 and SSI-5 score.

### 4.4. Changes in the amygdala RSFC after treatment and their relationship with the improvements in depressive symptoms and suicidal ideation

After ketamine treatment, the HAMD-17 score significantly decreased by 11.0 points (23.2 ± 4.6 vs. 12.2 ± 7.4, *p* < 0.001), and the SSI-5 score significantly decreased by 3.3 points (9.0 ± 3.5 vs. 6.0 ± 2.3, *p* < 0.001).

**FIGURE 3:**
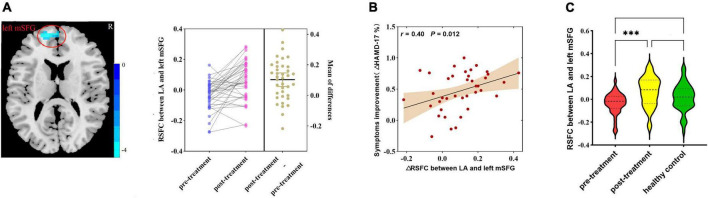
**(A)** Differences in RSFC between pre-treatment and post-treatment MDD patients (paired samples *t*-test, voxel-level *p* < 0.001, corrected by FWE; left amygdala cluster size threshold *k* ≥ 40). Color bar represents *t*-values. **(B)** Correlation analysis of changes in RSFC of the amygdala with the rate of reduction in HAMD-17 in MDD patients after ketamine treatment. **(C)** Differences in RSFC among pre-treatment, post-treatment MDD patients, and HCs (analysis of variance). RSFC, resting-state functional connectivity; MDD, major depressive disorder; HAMD-17, 17-item of Hamilton Depression Rating Scale; LA, left amygdala; mSFG, medial superior frontal gyrus; ΔHAMD-17%, HAMD-17 reduction rate. ^***^p < 0.001.

After ketamine treatment, the RSFC between the LA and the left medial superior frontal gyrus (mSFG) was strengthened (Figure 3A). The altered RSFC between the LA and the left mSFG [cluster size = 43; Montreal Neurological Institute (MNI) coordinates: *x* = −9, *y* = 60, *z* = 15; pFWE-corr = 0.020), a brain region that exhibited significant changes after ketamine treatment, was positively correlated with the HAMD-17 reduction rate (*r* = 0.40, *p* = 0.012, Figure 3B). But the altered RSFC between the LA and the left mSFG was not correlated with the SSI-5 reduction rate. There was no significant difference in RSFC of left LA - left mSFG between the pre-treatment MDD patients and HCs and between post-treatment MDD patients and HCs (Figure 3C). The RSFC of the RA did not exhibit significant changes after treatment.

### 4.5. Baseline amygdala RSFC predicted ketamine’s antidepressant and antisuicidal effects

**FIGURE 4:**
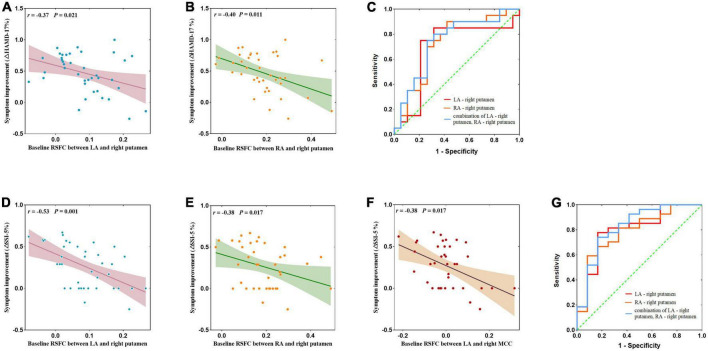
**(A,B,D–F)** Correlation between abnormal amygdala RSFC at baseline and the remission rate of depressive symptoms and suicidal symptoms after ketamine treatment and ROC curve analysis. **(C)** Neuroimaging predictors of treatment non-response, with the non-responders defined with <50% improvements in HAMD-17 obtained after the ketamine infusions. The area under the ROC curve was 0.689 (*p* = 0.043) for the RSFC of the LA – right putamen, with a sensitivity of 91.4%, and specificity of 59.1%; the area under the ROC curve was 0.726 (*p* = 0.016) for RA – right putamen connectivity, with a sensitivity of 82.4%, and specificity of 68.2%; and the area under the ROC curve was 0.739 (*p* = 0.011) for LA – right putamen connectivity and RA-right putamen connectivity, with a sensitivity of 75.0%, and specificity of 73.7%. **(G)** Non-responders defined with <50% improvements SSI-5 score obtained after the ketamine infusions. The area under the ROC curve was 0.799 (*p* = 0.003) for the RSFC of the LA – right putamen, with a sensitivity of 77.8%, and specificity of 83.3%; the area under the ROC curve was 0.787 (*p* = 0.005) for the RA – right putamen connectivity, with a sensitivity of 59.3%, and specificity of 91.7%; and the area under the ROC curve was 0.827 (*p* = 0.001) for LA – right putamen connectivity and RA – right putamen connectivity, with a sensitivity of 74.1%, and specificity of 83.3%. AUC, area under curve; LA, left amygdala; RA, right amygdala.

At baseline, the RSFC between the LA and the right putamen (*r* = −0.37, *p* = 0.021, Figure 4A) and the RSFC between the RA and the right putamen (*r* = −0.40, *p* = 0.011, Figure 4B) were negatively correlated with the reduction rate of HAMD-17 score, respectively. The RSFC between the LA and the right putamen (*r* = −0.53, *p* = 0.001, Figure 4D), the RSFC between the RA and the right putamen (*r* = −0.38, *p* = 0.017, Figure 4E) and the LA and right MCC (*r* = −0.38, *p* = 0.017, Figure 4F), were negatively correlated with the reduction rate of SSI-5, respectively.

For antidepressant response, ROC curves analysis was showed that the baseline amygdala RSFC was significant predictor, with the AUC of the LA – right putamen connectivity was 0.689 [95% confidence interval (CI), 0.504–0.875; *p* = 0.043], the AUC of the RA – right putamen connectivity was 0.726 (95% CI, 0.561–0.892; *p* = 0.016) and the AUC of combination of LA – right putamen connectivity and RA – right putamen connectivity was 0.739 (95% CI, 0.578–0.901; *p* = 0.011). See Figure 4C and Table 3 for details.

**TABLE 3:** Results of ROC curve analysis of antidepressant and antisuicidal response at 24 h after six ketamine infusions.

Independent variables	AUC	Cut-off	Sensibility	Specificity	Youden index
**Antidepressant response**					
LA – right putamen	0.689	0.085	0.750	0.789	0.539
RA – right putamen	0.726	0.136	0.900	0.579	0.479
Combination of LA – right putamen, RA – right putamen	0.739	0.513	0.750	0.737	0.487
**Antisuicidal response**					
LA – right putamen	0.799	0.066	0.778	0.833	0.611
RA – right putamen	0.787	0.197	0.593	0.917	0.510
Combination of LA – right putamen, RA – right putamen	0.827	0.664	0.741	0.833	0.574

AUC, area under curve; LA, left amygdala; RA, right amygdala.

For antisuicidal response, the AUC of the LA - right putamen connectivity was 0.799 (95% CI, 0.641 to 0.958; *p* = 0.003), and the AUC of the RA- right putamen connectivity was 0.787 (95% CI, 0.628 to 0.946; *p* = 0.005) and the AUC of combination of LA - right putamen connectivity and RA-right putamen connectivity was 0.827 (95% CI, 0.676 to 0.978; *p* = 0.001). See Figure 4G and Table 3 for details.

## 5. Discussion

In this study, we found that after ketamine treatment, the RSFC between the LA and the left mSFG of MDD patients enhanced significantly, and this change was positively correlated with the improvements in depressive symptoms. The baseline RSFC of the LA and right putamen, and the baseline RSFC of the RA and right putamen could predict antidepressant and the antisuicidal effects of ketamine.

Previous studies reported that the RSFC of the amygdala in MDD patients differ from that of HCs (Cheng et al., 2018; Tang et al., 2018), and our findings further validate this conclusion. Our study found that the strength of the RSFC between the amygdala and insula was associated with the severity of depression; the weaker the RSFC was, the more severe the depressive symptoms, consistent with previous study (Bebko et al., 2015). In addition, we found that the strength of the RSFC between amygdala and putamen, MCC and PCG were related to suicide, and these regions associated with mood and the risk of suicide (Straube and Miltner, 2011; van Heeringen et al., 2014; Gifuni et al., 2021). However, few studies have explored the relationship between the RSFC of the amygdala and suicidal ideation in MDD patients. One study reported that suicide was associated with the RSFC between the amygdala and the insula, middle temporal gyrus, and supraorbital frontal regions (Kang et al., 2017). Possible explanations for the lack of consistent findings were differences in the methods used to analyze the RSFC and the heterogeneity of subjects. In addition, the sample sizes of our study and the above studies was small; further investigations should increase the sample size.

In the present study, we found strengthened RSFC between the LA and left mSFG after ketamine treatment; these changes were positively correlated with the improvements in depressive symptoms. The mSFG is part of the medial PFC (mPFC), which is normally extensively associated with the amygdala and exerts top-down inhibitory control over its activity, thereby regulating emotional expression (Likhtik et al., 2014; Bukalo et al., 2015). Hyperactivity of the amygdala in MDD patients has been suggested to result from reduced inhibitory input. Although our study did not find a significant difference in the RSFC between the amygdala and mPFC between MDD patients and HCs, previous studies of MDD patients have found weakened RSFC in the amygdala and PFC (Dannlowski et al., 2009; Tang et al., 2013; Connolly et al., 2017). A previous study found that the RSFC between the amygdala and PFC was strengthened after treatment with conventional antidepressants and that the RSFC of the LA was more significantly strengthened than that of the RA (Chen et al., 2008). The effect of ketamine may be related to mPFC activity. One study reported that the concentration of glutamate and glutamine complex in mPFC of ketamine responder began to rise rapidly when ketamine was injected (Milak et al., 2016). Another study showed that subanesthetic doses of ketamine cause a glutamatergic burst in the mPFC and enhanced mPFC activity, whereas silencing the mPFC blocks the effects of ketamine (Fuchikami et al., 2015). Additionally, MDD patients exhibited reduced PFC volume, decreased neurotrophic factor release, and neuronal atrophy (Botteron et al., 2002; Duman and Monteggia, 2006; Duman and Li, 2012). Ketamine could promote the production and release of neurotrophic factors within the mPFC and restores its function (Li et al., 2010, 2011). Therefore, the strengthened RSFC between the LA and left mSFG after ketamine treatment further indicated that the antidepressant effect of ketamine may be related to enhanced PFC activity to regulate amygdala function.

Our study found that the RSFC between the amygdala and the right putamen may be a predictor of clinical response to ketamine treatment. MDD patients with greater decoupling of amygdala and right putamen may be the target population for ketamine treatment. The putamen is an important part of the striatum, a system that increases responses to negative emotions and is a core region of the reward network (Keren et al., 2018). Previous studies have demonstrated that loss of pleasure in MDD patients was associated with abnormal reward circuits. One study showed that glycolysis in the putamen of bipolar depressive patients with treatment-resistant was associated with reduced anhedonia and the anti-anhedonic effects of ketamine was due to ketamine-induced increases in glucose metabolism in the putamen and MCC (Lally et al., 2014). Moreover, Yang et al. (2017) found that female MDD patients had reduced amygdala and putamen volume and decreased RSFC between amygdala and putamen, which was similar to our results. In our study, we also found that the lower RSFC between amygdala and putamen was associated with stronger suicidal ideation, which may suggest that functional decoupling between amygdala and putamen may be related to the occurrence of suicidal ideation. Previous studies suggest that the putamen was also involved in suicidal ideation. Putamen volume was decreased in MDD patients with suicidal ideation (Dombrovski et al., 2012), and that lower putamen activation during motor tasks was associated with higher suicidal ideation in adult BD patients (Marchand et al., 2011). After ketamine treatment, putamen gray matter volume increased, and activity in brain regions such as the putamen and caudate nucleus increased (Rao et al., 2017; Gallay et al., 2021). Our findings may further confirm that the antidepressant and antisuicide effects of ketamine may be related to the functional changes of reward related brain regions.

In addition, our study found that RSFC between the amygdala and MCC was associated with improvement in suicidal ideation. MCC is also thought to be part of the reward circuit, involved in action reinforcement association and selection based on the rewarding or aversive nature of the underlying movement (Bracht et al., 2015). Animal studies have found that this amygdala – MCC pathway is associated with the influence of the amygdala’s facial processing subsystem on the perception of emotional facial expressions, such as fearful, sad, and happy expressions (Morecraft et al., 2007; Rolls, 2007; Grabenhorst and Rolls, 2011). Previous study has suggested that the sustained antidepressant effect of a low-dose ketamine infusion was mediated by increased activation of the supplementary motor area (SMA) and MCC; a single optimal dose of ketamine facilitated glutamatergic neurotransmission in the SMA and MCC, activating both regions, whereas a greater increase in the activation of MCC was associated with a reduction in depressive symptoms (Chen et al., 2018). Except for ketamine, other drug also seem to target the RSFC between the amygdala and MCC. One study showed that after 8-weeks of quetiapine treatment, decreased HAMD scores in anxiety depressive patients were associated with increased RSFC in the amygdala-MCC and amygdala-precuneus (Altinay et al., 2016). Our results reveal a connection between the amygdala and MCC that may also be a target for ketamine treatment.

This study had several limitations. First, the sample size was small. Since MDD patients commonly experience symptoms of anxiety, they are prone to irritability during scans, causing excessive head movement, which affects the acquisition of high-quality data. Second, due to the open-label design and the lack of placebo-treated controls, the influence of temporal effects on functional changes in the brain cannot be excluded. Third, RSFC describes the temporal correlation of blood signals between brain regions, that is, the synchronization of functional activity in related brain regions; it cannot determine anatomical directionality or causal relationships. Fourth, as all MDD patients were not restricted in terms of the use of psychiatric drugs throughout the study, we cannot rule out a group effect due to medication. Fifth, we focused on the amygdala and explored its RSFC with the whole brain, but the results indicated that the amygdala is not a single homogeneous structure and exhibits structural and functional subdivisions; thus, further exploration of the subregions of the amygdala is needed.

## 6. Conclusion

Short-term repeated use of ketamine may modulate the RSFC of the amygdala in MDD patients. The mechanism of improving depressive symptoms by ketamine may be related to it is regulation of the RSFC of amygdala. The combination baseline RSFC of bilateral amygdala and right putamen may be a predictor of the response of ketamine’s antidepressant and antisuicidal treatment.

## Data availability statement

The raw data supporting the conclusions of this article will be made available by the authors, without undue reservation.

## Ethics statement

The studies involving human participants were reviewed and approved by the Ethics Committee of Guangzhou Hui’ai Hospital. The patients/participants provided their written informed consent to participate in this study.

## Author contributions

HL: investigation, formal analysis, writing—original drafting, and visualization. CW, XL, and WL: validation and investigation. FZ, LF, and YY: investigation. YN and YZ: conceptualization, supervision, and writing—review and editing. All authors contributed to the article and approved the submitted version.

## Footnotes

1
http://rfmri.org/DPARSF

2
https://www.fil.ion.ucl.ac.uk/spm/

## References

[B1] Aan H. R. M. Collins K. A. Murrough J. W. Perez A. M. Reich D. L. Charney D. S. (2010). Safety and efficacy of repeated-dose intravenous ketamine for treatment-resistant depression. *Biol. Psychiatry* 67 139–145. 10.1016/j.biopsych.2009.08.038 19897179

[B2] Altinay M. Karne H. Beall E. Anand A. (2016). Quetiapine extended release open-label treatment associated changes in amygdala activation and connectivity in anxious depression: An fMRI study. *J. Clin. Psychopharmacol.* 36 562–571. 10.1097/JCP.0000000000000600 27768670

[B3] Bebko G. Bertocci M. Chase H. Dwojak A. Bonar L. Almeida J. (2015). Decreased amygdala-insula resting state connectivity in behaviorally and emotionally dysregulated youth. *Psychiatry Res.* 231 77–86. 10.1016/j.pscychresns.2014.10.015 25433424 PMC4272653

[B4] Botteron K. N. Raichle M. E. Drevets W. C. Heath A. C. Todd R. D. (2002). Volumetric reduction in left subgenual prefrontal cortex in early onset depression. *Biol. Psychiatry* 51 342–344. 10.1016/S0006-3223(01)01280-X 11958786

[B5] Bracht T. Linden D. Keedwell P. (2015). A review of white matter microstructure alterations of pathways of the reward circuit in depression. *J. Affect. Disord.* 187 45–53. 10.1016/j.jad.2015.06.041 26318270

[B6] Bukalo O. Pinard C. R. Silverstein S. Brehm C. Hartley N. Whittle N. (2015). Prefrontal inputs to the amygdala instruct fear extinction memory formation. *Sci. Adv.* 1:e1500251. 10.1126/sciadv.1500251 26504902 PMC4618669

[B7] Chen C. Suckling J. Ooi C. Fu C. H. Williams S. C. Walsh N. D. (2008). Functional coupling of the amygdala in depressed patients treated with antidepressant medication. *Neuropsychopharmacology* 33 1909–1918. 10.1038/sj.npp.1301593 17987064

[B8] Chen M. Li C. Lin W. Hong C. Tu P. Bai Y. (2018). Persistent antidepressant effect of low-dose ketamine and activation in the supplementary motor area and anterior cingulate cortex in treatment-resistant depression: A randomized control study. *J. Affect. Disord.* 225 709–714. 10.1016/j.jad.2017.09.008 28922734

[B9] Cheng W. Rolls E. T. Qiu J. Xie X. Lyu W. Li Y. (2018). Functional connectivity of the human amygdala in health and in depression. *Soc. Cogn. Affect. Neurosci.* 13 557–568. 10.1093/scan/nsy032 29767786 PMC6022538

[B10] Connolly C. G. Ho T. C. Blom E. H. LeWinn K. Z. Sacchet M. D. Tymofiyeva O. (2017). Resting-state functional connectivity of the amygdala and longitudinal changes in depression severity in adolescent depression. *J. Affect. Disord.* 207 86–94. 10.1016/j.jad.2016.09.026 27716542 PMC5149416

[B11] Dannlowski U. Ohrmann P. Konrad C. Domschke K. Bauer J. Kugel H. (2009). Reduced amygdala-prefrontal coupling in major depression: Association with MAOA genotype and illness severity. *Int. J. Neuropsychopharmacol.* 12 11–22. 10.1017/S1461145708008973 18544183

[B12] Davis M. Whalen P. J. (2001). The amygdala: Vigilance and emotion. *Mol. Psychiatry* 6 13–34. 10.1038/sj.mp.4000812 11244481

[B13] Dombrovski A. Y. Siegle G. J. Szanto K. Clark L. Reynolds C. F. Aizenstein H. (2012). The temptation of suicide: Striatal gray matter, discounting of delayed rewards, and suicide attempts in late-life depression. *Psychol. Med.* 42 1203–1215. 10.1017/S0033291711002133 21999930 PMC3368587

[B14] Duman R. S. Li N. (2012). A neurotrophic hypothesis of depression: Role of synaptogenesis in the actions of NMDA receptor antagonists. *Philos. Trans. R. Soc. Lond. B Biol. Sci.* 367 2475–2484. 10.1098/rstb.2011.0357 22826346 PMC3405673

[B15] Duman R. S. Monteggia L. M. (2006). A neurotrophic model for stress-related mood disorders. *Biol. Psychiatry* 59 1116–1127. 10.1016/j.biopsych.2006.02.013 16631126

[B16] Engman J. Linnman C. Dijk K. R. Milad M. R. (2016). Amygdala subnuclei resting-state functional connectivity sex and estrogen differences. *Psychoneuroendocrinology* 63 34–42. 10.1016/j.psyneuen.2015.09.012 26406106

[B17] Ferrari A. J. Charlson F. J. Norman R. E. Patten S. B. Freedman G. Murray C. J. (2013). Burden of depressive disorders by country, sex, age, and year: Findings from the global burden of disease study 2010. *PLoS Med.* 10:e1001547. 10.1371/journal.pmed.1001547 24223526 PMC3818162

[B18] Fox M. D. Zhang D. Snyder A. Z. Raichle M. E. (2009). The global signal and observed anticorrelated resting state brain networks. *J. Neurophysiol.* 101 3270–3283. 10.1152/jn.90777.2008 19339462 PMC2694109

[B19] Fuchikami M. Thomas A. Liu R. Wohleb E. S. Land B. B. DiLeone R. J. (2015). Optogenetic stimulation of infralimbic PFC reproduces ketamine’s rapid and sustained antidepressant actions. *Proc. Natl. Acad. Sci. U.S.A.* 112 8106–8111. 10.1073/pnas.1414728112 26056286 PMC4491758

[B20] Gallay C. C. Forsyth G. Can A. T. Dutton M. Jamieson D. Jensen E. (2021). Six-week oral ketamine treatment for chronic suicidality is associated with increased grey matter volume. *Psychiatry Res. Neuroimaging* 317:111369. 10.1016/j.pscychresns.2021.111369 34461430

[B21] Gifuni A. J. Chakravarty M. M. Lepage M. Ho T. C. Geoffroy M. Lacourse E. (2021). Brain cortical and subcortical morphology in adolescents with depression and a history of suicide attempt. *J. Psychiatry Neurosci.* 46 E347–E357. 10.1503/jpn.200198 33961355 PMC8327980

[B22] Grabenhorst F. Rolls E. T. (2011). Value, pleasure and choice in the ventral prefrontal cortex. *Trends Cogn. Sci.* 15 56–67. 10.1016/j.tics.2010.12.004 21216655

[B23] Kang S. Na K. Choi J. Kim J. Son Y. Lee Y. J. (2017). Resting-state functional connectivity of the amygdala in suicide attempters with major depressive disorder. *Prog. Neuropsychopharmacol. Biol. Psychiatry* 77 222–227. 10.1016/j.pnpbp.2017.04.029 28445688

[B24] Keren H. O’Callaghan G. Vidal-Ribas P. Buzzell G. A. Brotman M. A. Leibenluft E. (2018). Reward processing in depression: A conceptual and meta-analytic review across fMRI and EEG studies. *Am. J. Psychiatry* 175 1111–1120. 10.1176/appi.ajp.2018.17101124 29921146 PMC6345602

[B25] Lally N. Nugent A. C. Luckenbaugh D. A. Ameli R. Roiser J. P. Zarate C. A. (2014). Anti-anhedonic effect of ketamine and its neural correlates in treatment-resistant bipolar depression. *Transl. Psychiatry* 4:e469. 10.1038/tp.2014.105 25313512 PMC4350513

[B26] Li N. Lee B. Liu R. Banasr M. Dwyer J. M. Iwata M. (2010). mTOR-dependent synapse formation underlies the rapid antidepressant effects of NMDA antagonists. *Science* 329 959–964. 10.1126/science.1190287 20724638 PMC3116441

[B27] Li N. Liu R. Dwyer J. M. Banasr M. Lee B. Son H. (2011). Glutamate N-methyl-D-aspartate receptor antagonists rapidly reverse behavioral and synaptic deficits caused by chronic stress exposure. *Biol. Psychiatry* 69 754–761. 10.1016/j.biopsych.2010.12.015 21292242 PMC3068225

[B28] Likhtik E. Stujenske J. M. Topiwala M. A. Harris A. Z. Gordon J. A. (2014). Prefrontal entrainment of amygdala activity signals safety in learned fear and innate anxiety. *Nat. Neurosci.* 17 106–113. 10.1038/nn.3582 24241397 PMC4035371

[B29] Liu J. Fang J. Wang Z. Rong P. Hong Y. Fan Y. (2016). Transcutaneous vagus nerve stimulation modulates amygdala functional connectivity in patients with depression. *J. Affect. Disord.* 205 319–326. 10.1016/j.jad.2016.08.003 27559632

[B30] Marcantoni W. S. Akoumba B. S. Wassef M. Mayrand J. Lai H. Richard-Devantoy S. (2020). A systematic review and meta-analysis of the efficacy of intravenous ketamine infusion for treatment resistant depression: January 2009–January 2019. *J. Affect. Disord.* 277 831–841. 10.1016/j.jad.2020.09.007 33065824

[B31] Marchand W. R. Lee J. N. Garn C. Thatcher J. Gale P. Kreitschitz S. (2011). Striatal and cortical midline activation and connectivity associated with suicidal ideation and depression in bipolar II disorder. *J. Affect. Disord.* 133 638–645. 10.1016/j.jad.2011.04.039 21621263

[B32] McGrath P. J. Stewart J. W. Fava M. Trivedi M. Wisniewski S. Nierenberg A. (2006). Tranylcypromine versus venlafaxine plus mirtazapine following three failed antidepressant medication trials for depression: A STAR*D report. *Am. J. Psychiatry* 163 1531–1541. 10.1176/ajp.2006.163.9.1531 16946177

[B33] Milak M. S. Proper C. J. Mulhern S. T. Parter A. L. Kegeles L. S. Ogden R. T. (2016). A pilot in vivo proton magnetic resonance spectroscopy study of amino acid neurotransmitter response to ketamine treatment of major depressive disorder. *Mol. Psychiatry* 21 320–327. 10.1038/mp.2015.83 26283639 PMC4758914

[B34] Morecraft R. J. McNeal D. W. Stilwell-Morecraft K. S. Gedney M. Ge J. Schroeder C. M. (2007). Amygdala interconnections with the cingulate motor cortex in the rhesus monkey. *J. Comp. Neurol.* 500 134–165. 10.1002/cne.21165 17099887

[B35] Mrazek D. A. Hornberger J. C. Altar C. A. Degtiar I. (2014). A review of the clinical, economic, and societal burden of treatment-resistant depression: 1996-2013. *Psychiatr. Serv.* 65 977–987. 10.1176/appi.ps.201300059 24789696

[B36] Murrough J. W. Perez A. M. Pillemer S. Stern J. Parides M. K. Rot M a (2013). Rapid and longer-term antidepressant effects of repeated ketamine infusions in treatment-resistant major depression. *Biol. Psychiatry* 74 250–256. 10.1016/j.biopsych.2012.06.022 22840761 PMC3725185

[B37] Phelps E. A. LeDoux J. E. (2005). Contributions of the amygdala to emotion processing: From animal models to human behavior. *Neuron* 48 175–187. 10.1016/j.neuron.2005.09.025 16242399

[B38] Price J. L. (2003). Comparative aspects of amygdala connectivity. *Ann. N. Y. Acad. Sci.* 985 50–58. 10.1111/j.1749-6632.2003.tb07070.x 12724147

[B39] Rao J. Liu Z. Zhao C. Wei R. Zhao W. Tian P. (2017). Ketamine changes the local resting-state functional properties of anesthetized-monkey brain. *Magn. Reson. Imaging* 43 144–150. 10.1016/j.mri.2017.07.025 28755862

[B40] Rolls E. T. (2007). The representation of information about faces in the temporal and frontal lobes. *Neuropsychologia* 45 124–143. 10.1016/j.neuropsychologia.2006.04.019 16797609

[B41] Straube T. Miltner W. H. (2011). Attention to aversive emotion and specific activation of the right insula and right somatosensory cortex. *Neuroimage* 54 2534–2538. 10.1016/j.neuroimage.2010.10.010 20946962

[B42] Tang S. Lu L. Zhang L. Hu X. Bu X. Li H. (2018). Abnormal amygdala resting-state functional connectivity in adults and adolescents with major depressive disorder: A comparative meta-analysis. *EBioMedicine* 36 436–445. 10.1016/j.ebiom.2018.09.010 30316866 PMC6197798

[B43] Tang Y. Kong L. Wu F. Womer F. Jiang W. Cao Y. (2013). Decreased functional connectivity between the amygdala and the left ventral prefrontal cortex in treatment-naive patients with major depressive disorder: A resting-state functional magnetic resonance imaging study. *Psychol. Med.* 43 1921–1927. 10.1017/S0033291712002759 23194671

[B44] Trivedi M. H. Rush A. J. Wisniewski S. R. Nierenberg A. A. Warden D. Ritz L. (2006). Evaluation of outcomes with citalopram for depression using measurement-based care in STAR*D: Implications for clinical practice. *Am. J. Psychiatry* 163 28–40. 10.1176/appi.ajp.163.1.28 16390886

[B45] Tzourio-Mazoyer N. Landeau B. Papathanassiou D. Crivello F. Etard O. Delcroix N. (2002). Automated anatomical labeling of activations in SPM using a macroscopic anatomical parcellation of the MNI MRI single-subject brain. *Neuroimage* 15 273–289. 10.1006/nimg.2001.0978 11771995

[B46] van Heeringen K. Bijttebier S. Desmyter S. Vervaet M. Baeken C. (2014). Is there a neuroanatomical basis of the vulnerability to suicidal behavior? A coordinate-based meta-analysis of structural and functional MRI studies. *Front. Hum. Neurosci.* 8:824. 10.3389/fnhum.2014.00824 25374525 PMC4205829

[B47] Wilkinson S. T. Ballard E. D. Bloch M. H. Mathew S. J. Murrough J. W. Feder A. (2018). The effect of a single dose of intravenous ketamine on suicidal ideation: A systematic review and individual participant data meta-analysis. *Am. J. Psychiatry* 175 150–158. 10.1176/appi.ajp.2017.17040472 28969441 PMC5794524

[B48] Yang J. Yin Y. Svob C. Long J. He X. Zhang Y. (2017). Amygdala atrophy and its functional disconnection with the cortico-striatal-pallidal-thalamic circuit in major depressive disorder in females. *PLoS One* 12:e0168239. 10.1371/journal.pone.0168239 28107446 PMC5249227

[B49] Zhang R. Zhang L. Wei S. Wang P. Jiang X. Tang Y. (2020). Increased amygdala-paracentral lobule/precuneus functional connectivity associated with patients with mood disorder and suicidal behavior. *Front. Hum. Neurosci.* 14:585664. 10.3389/fnhum.2020.585664 33519398 PMC7843440

[B50] Zheng W. Zhou Y. Liu W. Wang C. Zhan Y. Li H. (2018). Rapid and longer-term antidepressant effects of repeated-dose intravenous ketamine for patients with unipolar and bipolar depression. *J. Psychiatr. Res.* 106 61–68. 10.1016/j.jpsychires.2018.09.013 30278319

[B51] Zheng W. Zhou Y. Liu W. Wang C. Zhan Y. Li H. (2019). Investigation of medical effect of multiple ketamine infusions on patients with major depressive disorder. *J. Psychopharmacol.* 33 494–501. 10.1177/0269881119827811 30789302

[B52] Zhou Y. Wu F. Liu W. Zheng W. Wang C. Zhan Y. (2020). Volumetric changes in subcortical structures following repeated ketamine treatment in patients with major depressive disorder: A longitudinal analysis. *Transl. Psychiatry* 10:264. 10.1038/s41398-020-00945-9 32747631 PMC7400625

[B53] Zhou Y. Zheng W. Liu W. Wang C. Zhan Y. Li H. (2018a). Antidepressant effect of repeated ketamine administration on kynurenine pathway metabolites in patients with unipolar and bipolar depression. *Brain Behav. Immun.* 74 205–212. 10.1016/j.bbi.2018.09.007 30213652

[B54] Zhou Y. Zheng W. Liu W. Wang C. Zhan Y. Li H. (2018b). Neurocognitive effects of six ketamine infusions and the association with antidepressant response in patients with unipolar and bipolar depression. *J. Psychopharmacol.* 32 1118–1126. 30260273 10.1177/0269881118798614

